# Hydroxyapatite or Fluorapatite—Which Bioceramic Is Better as a Base for the Production of Bone Scaffold?—A Comprehensive Comparative Study

**DOI:** 10.3390/ijms24065576

**Published:** 2023-03-14

**Authors:** Paulina Kazimierczak, Joanna Wessely-Szponder, Krzysztof Palka, Adriana Barylyak, Viktor Zinchenko, Agata Przekora

**Affiliations:** 1 Independent Unit of Tissue Engineering and Regenerative Medicine, Medical University of Lublin, Chodzki 1 Street, 20-093 Lublin, Poland; agata.przekora@umlub.pl; 2 Sub-Department of Pathophysiology, Department of Preclinical Veterinary Sciences, University of Life Sciences in Lublin, Akademicka 13 Street, 20-950 Lublin, Poland; joanna.wessely@up.lublin.pl; 3 Department of Materials Engineering, Lublin University of Technology, Nadbystrzycka 36 Street, 20-618 Lublin, Poland; k.palka@pollub.pl; 4 Department of Therapeutic Dentistry, Danylo Halytsky Lviv National Medical University, Pekarska 69 Street, 79010 Lviv, Ukraine; adriana.barylyak5@gmail.com; 5 Department of Chemistry of Functional Inorganic Materials, Bogatsky Physico-Chemical Institute of the National Academy of Sciences of Ukraine, Lustdorfskaya Doroga 86, 65080 Odessa, Ukraine; vfzinchenko@ukr.net

**Keywords:** calcium phosphates, scaffold, biomaterial, compressive strength, bioactivity, biodegradation, biocompatibility, osteoblast, osteogenic differentiation, cytotoxicity

## Abstract

Hydroxyapatite (HAP) is the most common calcium phosphate ceramic that is used in biomedical applications, e.g., as an inorganic component of bone scaffolds. Nevertheless, fluorapatite (FAP) has gained great attention in the area of bone tissue engineering in recent times. The aim of this study was a comprehensive comparative evaluation of the biomedical potential of fabricated HAP- and FAP-based bone scaffolds, to assess which bioceramic is better for regenerative medicine applications. It was demonstrated that both biomaterials had a macroporous microstructure, with interconnected porosity, and were prone to slow and gradual degradation in a physiological environment and in acidified conditions mimicking the osteoclast-mediated bone resorption process. Surprisingly, FAP-based biomaterial revealed a significantly higher degree of biodegradation than biomaterial containing HAP, which indicated its higher bioabsorbability. Importantly, the biomaterials showed a similar level of biocompatibility and osteoconductivity regardless of the bioceramic type. Both scaffolds had the ability to induce apatite formation on their surfaces, proving their bioactive property, that is crucial for good implant osseointegration. In turn, performed biological experiments showed that tested bone scaffolds were non-toxic and their surfaces promoted cell proliferation and osteogenic differentiation. Moreover, the biomaterials did not exert a stimulatory effect on immune cells, since they did not generate excessive amounts of reactive oxygen species (ROS) and reactive nitrogen species (RNS), indicating a low risk of inflammatory response after implantation. In conclusion, based on the obtained results, both FAP- and HAP-based scaffolds have an appropriate microstructure and high biocompatibility, being promising biomaterials for bone regeneration applications. However, FAP-based biomaterial has higher bioabsorbability than the HAP-based scaffold, which is a very important property from the clinical point of view, because it enables a progressive replacement of the bone scaffold with newly formed bone tissue.

## 1. Introduction

Bone is the second most common transplant tissue. The gold standard of bone grafting is harvesting autologous tissue from the iliac crest. However, autograft harvesting is characterized by some drawbacks, such as chronic pain at the donor site, risk of infection, and haematoma formation. In turn, allografts, derived from cadavers or living donors of the same species, carry a risk of immune reaction and the possibility of infection or disease transmission [1]. Thus, the ageing population, and the increasing incidence of bone disorders, have resulted in the need to develop new effective clinical therapies. Bone tissue engineering strategies are considered as alternatives to conventional bone grafts, for repairing bone defects [2]. A common approach to bone tissue engineering involves applying a three-dimensional porous scaffold, bone-forming cells, signaling factors, and in vitro culture using e.g., bioreactor systems [2,3]. The ideal scaffold should support cell attachment, adhesion, proliferation, and osteogenic differentiation. It should also possess mechanical properties comparable to native bone. Moreover, the biomaterial should ideally have a degradation rate that matches the native regenerative rates of bones, so as to support efficient bone regeneration [4]. Bone is a heterogeneous tissue, comprising a mineral component, hydroxyapatite (Ca_10_(PO_4_)_6_(OH)_2_), organic components (type I collagen, non-collagenous proteins, lipids), and water. Thus, to mimic the microstructure of bone, composite biomaterials containing polymers (natural or synthetic) and bioceramics (e.g., calcium phosphates) are frequently synthesized, to potentially achieve greater bioactivity and biocompatibility [5]. It is known that bioceramic type biomaterials have good osteoconductive and osteoinductive properties, but possess low mechanical strength, high brittleness, and a slow resorption rate. In turn, combining ceramics with polymers produces biomaterials with good mechanical properties [6]. Thus, the development of ceramic–polymer composites has a growing interest in the bone tissue engineering field.

Synthetic hydroxyapatite (HAP) is the most common calcium phosphate bioceramic that is used in biomedical applications (e.g., bone scaffold, bone filler, implant coating, drug delivery system) because of its similar chemical composition (calcium to phosphorus (Ca:P) ratio fixed at 1.67) to natural HAP, found in human bone and teeth [7,8,9]. HAP can be harvested from natural sources, such as corals or animal bones, or synthesized with chemical precursors, including Ca and P, using various methods, such as dry, wet, and thermal fabrication methods (singly or combinations thereof) [6,7]. Each of these methods may yield HAP with different characteristics, like bioactivity, bioabsorbability, and biocompatibility, owing to the various obtained crystalline phases, sizes, and morphologies of the calcium phosphates [7]. It is worth emphasizing that most commercially available HAPs are obtained at high temperatures (≥900 °C), by a sintering process [10]. HAP sintered at high temperatures displays high biocompatibility and structural similarity with the native bone mineral, however, it exhibits a low specific surface area (2–5 m^2^/g), reduced bioactivity, and poor solubility, and consequently low bioresorption. Bioresorption is the process in which the biomaterial is gradually degraded in the body over time, either by osteoclasts or by dissolution, to be replaced with the natural host tissue. Thus, “super stable” bioceramics are unable to undergo natural bone remodeling and remain at the site of implantation for years, delaying osseointegration [10,11,12]. In turn, HAP sintered at lower temperatures shows a higher specific surface area, that enhances access to body fluids and bone remodeling, improving bioabsorbability [11]. Nevertheless, it has also been reported that bioceramics sintered at low temperatures display high ion reactivity, which may significantly change the ionic composition in the microenvironment, causing a cytotoxic effect [13,14].

Various ions, such as magnesium (Mg^2+^), zinc (Zn^2+^), silicon (Si^2+^), strontium (Sr^2+^), copper (Cu^2+^), cobalt (Co^2+^), lithium (Li^+^), and fluoride (F^−^), can be substituted into the microstructure of HAP, to improve its biological and physicochemical properties [15]. For example, Sr-HAP coatings have been shown to induce the osteogenic differentiation of mesenchymal stem cells [16], likewise, Zn-HAP coatings stimulated mesenchymal stem cell differentiation into the osteoblasts and reduced the osteoclastic activity [17]. Chen et al. (2019) [18] showed that the substitution of Ca with Mg ions reduced the crystallinity of HAP crystals. In turn, 3D-printed Mg-HAP composite scaffolds showed better cell adhesion and proliferation and increased osteogenic-related gene expression. Recently, fluorapatite (FAP; Ca_10_(PO_4_)_6_F_2_) has attracted attention in the area of bone tissue regeneration and dental applications. Borkowski et al. (2021) [19] showed that a FAP-based scaffold was non-toxic and stimulated the adhesion of osteoblasts. Moreover, some studies showed that F^−^, released from FAP-based biomaterials, stimulated the proliferation and differentiation of osteoblasts [20,21] and supported the bone mineralization process [20]. Moreover, F^−^ ions may be helpful during osteoporosis treatment, by increasing bone mass [22]. Nevertheless, FAP is characterized by greater chemical stability and a lower bioresorption rate than HAP, making it less attractive for bone tissue engineering applications. It has been reported that the substitution of OH^−^ with F^−^ in the apatite ceramic may lower its solubility, hindering its replacement with newly formed tissue after implantation [23].

Many studies have reported physicochemical analysis and/or biological properties of HAP and FAP (either alone or as biomaterials, formed by combining them with other bioceramics or polymers). Nevertheless, very few reports have presented a comprehensive comparison of characteristics between HAP- and FAP-based biomaterials, to assess their potential for biomedical applications. In this study, HAP and FAP produced according to Ukraine patent no. 56202, were used for the production of bone scaffolds. HAP- and FAP-based biomaterials were synthesized according to the procedure described in the Polish patent no. 235822. The newly developed and patented synthesis method, combining a foaming agent with freeze-drying, allows the production of highly macroporous bone scaffolds, that may find potential applications in cancellous bone regeneration [24]. A comparative biological evaluation of the fabricated bioceramic-based bone scaffolds was performed by cytotoxicity, cell proliferation, and osteogenic differentiation tests, using human osteoblasts. Moreover, reactive oxygen species (ROS) and reactive nitrogen species (RNS) generation, by human immune cells, was assessed. Additionally, microstructure characterization, the mechanical properties, the biodegradation rate, and the apatite-forming ability on the surface of biomaterials, were estimated. Thus, results obtained within this study may give a new view on the appropriate selection of the apatite component, during biomaterial production for biomedical applications.

## 2. Results and Discussion

### 2.1. Characterization of Fabricated Biomaterials

**Figure 1:**
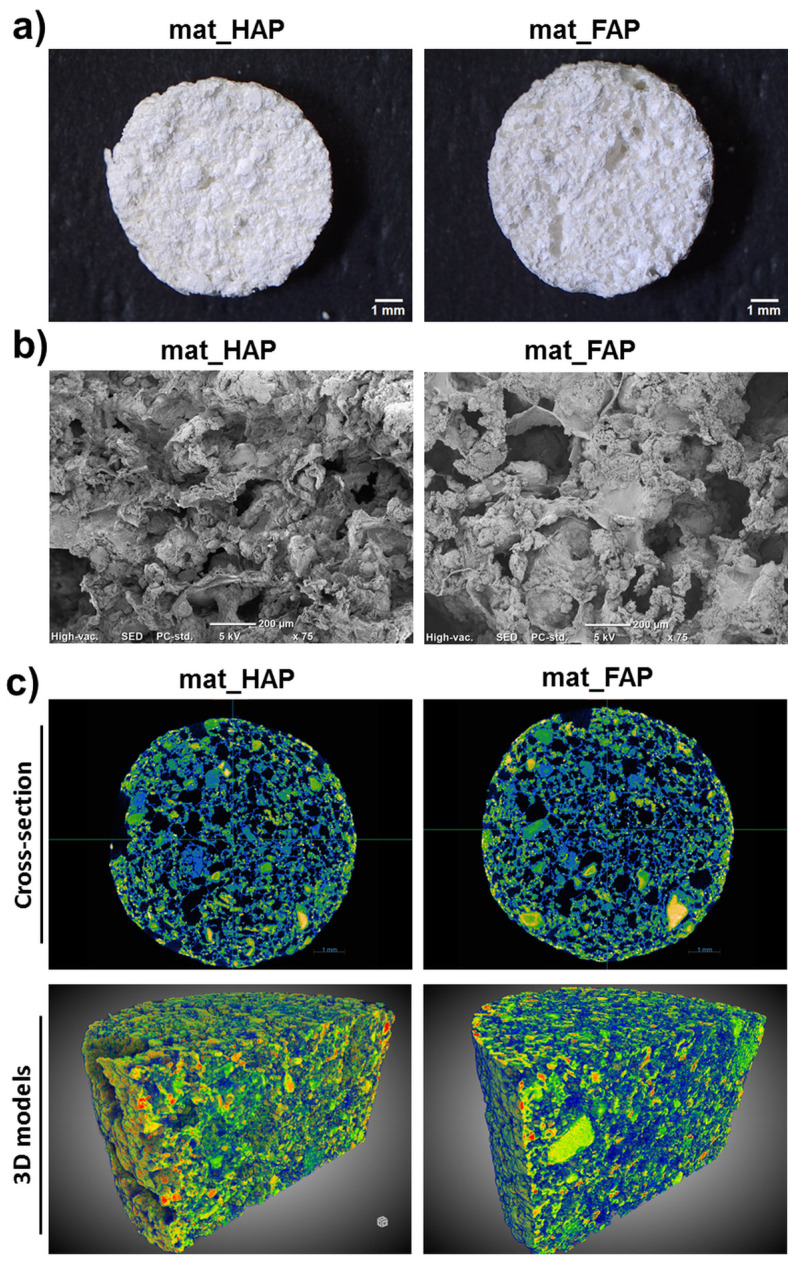
Microstructure evaluation of the apatite-based biomaterials by (**a**) stereoscopic microscope, (**b**) scanning electron microscope (SEM), and (**c**) microCT (yellow/green—ceramic; blue—polysaccharide matrix; black color on cross-section images—air voids).

The biomaterials for bone regeneration should possess not only suitable mechanical properties, but should also have optimal porosity, enabling appropriate gas and fluid transport through the whole scaffold, as well as providing sufficient space for the ingrowth of new bone [25]. Thus, the choice of a suitable production technique to produce three-dimensional (3D) structures, is a crucial step during the scaffold fabrication process. In this study, it was shown that it is possible to combine two simple fabrication methods, freeze-drying and gas-foaming, to fabricate macroporous scaffolds (Figure 1a,b). The fabricated biomaterials not only had a porous microstructure, but also rough and ragged surfaces. It is worth emphasizing that the surface roughness enhances the protein adsorption ability of the biomaterials, and thereby provides more effective cell attachment [25]. Moreover, visualization of the biomaterials’ microstructure, by micro-CT, demonstrated the presence of a well-interconnected pores network (Figure 1c). In turn, the quantitative evaluation of the biomaterials’ porosity, showed that mat_FAP was characterized by a significantly higher total porosity (47.52 ± 2.95%) than mat_HAP (37.52 ± 1.87%) (Table 1). Importantly, mat_FAP also exhibited significantly higher open porosity compared to mat_HAP. It should be noted, that open porosity, with interconnected pores, is the most important factor for rapid osseointegration of the implant with host tissue, enabling the formation of blood vessels within the biomaterial and better bone ingrowth [24]. It may be concluded, that the observed differences in the biomaterials’ porosity resulted from the particle size (HAP = 581 nm and FAP = 484 nm) and morphology of the fabricated apatites. Wojnarowska-Nowak et al. (2017) [26] performed an analysis of HAP and FAP by the transmission electron microscope technique, demonstrating that crystals of FAP had more elongated morphology compared to HAP, characterized by a spherical shape. Moreover, there are some reports proving the impact of apatite morphology on its interaction with the organic component of the scaffold, as well as its physicochemical and biological properties [26,27,28].

The compressive strength values determined for the fabricated biomaterials were as follows: 3.01 ± 0.59 MPa for mat_HAP and 2.58 ± 0.75 MPa for mat_FAP (Table 1). It was observed that the mechanical strength decreased with increasing porosity of the scaffold, which is a commonly observed phenomenon in the materials science field. The Young’s modulus values determined for the biomaterials were low: 19.14 ± 13.45 MPa for mat_HAP and 19.08 ± 10.38 MPa for mat_FAP (Table 1), proving their great elasticity. The Young’s modulus value of human cancellous bone averages between 0.1–0.5 GPa, whereas the compressive strength value fluctuates between 1.9–12 MPa [29], indicating the fabricated scaffolds were in the lower range of the compressive strength value of cancellous bone. Thus, considering also their low Young’s modulus values, they may find applications in non-load bearing implantation areas or in combination with wires, plates, or screws.

### 2.2. Apatite-Forming Ability

**Figure 2:**
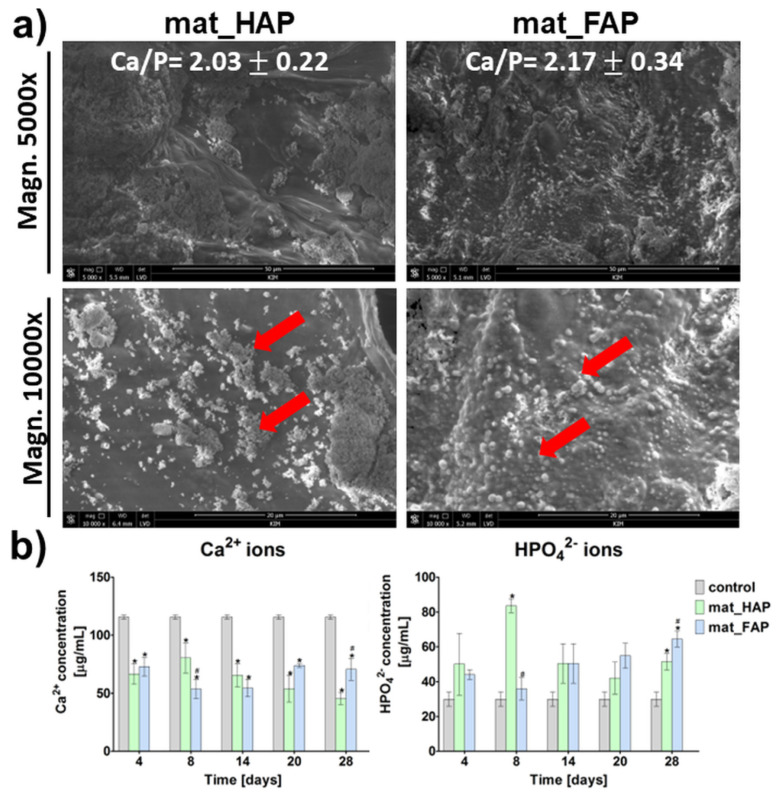
Apatite-forming ability of biomaterials: (**a**) SEM images of biomaterials’ surfaces after soaking in a simulated body fluid (SBF) for 28 days (red arrows indicate apatite crystals; Ca/P atomic ratio was calculated for apatite crystals), (**b**) changes in Ca^2+^ and HPO_4_^2−^ concentrations in SBF, during incubation with biomaterials (* statistically significant results compared to control SBF; *p* < 0.05, unpaired *t*-test).

The ability of biomaterials to form apatite crystals on their surfaces, is called bioactivity. It is a very important feature, since it provides good bonding with the surrounding bone tissue after implantation, facilitating the osseointegration process [30]. The in vitro apatite-forming ability of the bone scaffolds may be estimated by immersing them in simulated body fluid (SBF), with ion concentrations similar to those in blood plasma [31]. In this study, both of the fabricated biomaterials had the ability to induce apatite formation on their surfaces, after a 28-day incubation in SBF (Figure 2a). The apatite crystals formed had globular and hemispherical morphology (apatite crystals were indicated by red arrows on the SEM images). EDS analysis showed that the observed crystals were calcium phosphates, with Ca/P atomic ratios equal to 2.03 ± 0.22 and 2.17 ± 0.34, in the case of mat_HAP and mat_FAP, respectively. The stoichiometric value of the Ca/P atomic ratio for natural HAP is 1.67 [31]. Thus, the surfaces of mat_HAP and mat_FAP had the ability to form an amorphous phase of Ca-rich calcium phosphates. Nevertheless, it has been reported that, over time, the amorphous calcium phosphates have the ability to transform into apatites, with a Ca/P atomic ratio of 1.67 [32]. In turn, Figure 2b presents changes in Ca^2+^ and HPO_4_^2−^ concentrations in SBF, during incubation with the biomaterials. The obtained results were consistent with the EDS analysis of the apatite crystals after a 28-day incubation of the biomaterials in SBF, high uptake of Ca^2+^ ions by mat_HAP and mat_FAP was observed, which indicated the formation of Ca-rich amorphous calcium phosphates. Xie et al. [33] reported that a decrease in Ca^2+^ ions during the incubation of samples in SBF, may result either from electrostatic interaction between ions and the charged surface of the biomaterial and related apatite nucleation, or from ion exchange events. In turn, mat_HAP and mat_FAP caused an increase in HPO_4_^2−^ ions in SBF, compared to the control SBF, which probably resulted from the dissolution of biomaterials. The results obtained within this study are partly in agreement with the study of Borkowski et al. (2020) [34], who showed that HAP and FAP sintered at lower temperatures, were characterized by a high adsorbing capacity of Ca^2+^ and HPO_4_^2−^ ions from SBF.

### 2.3. Biodegradation Assessment

**Figure 3:**
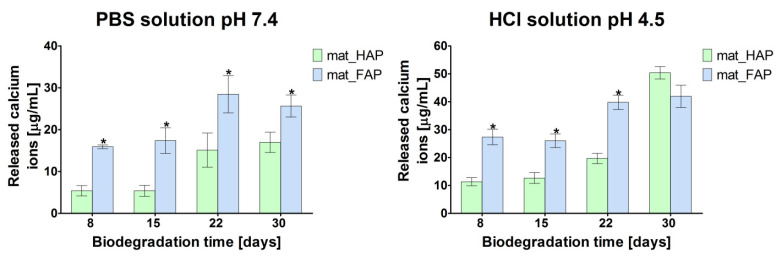
Evaluation of biomaterials’ biodegradation in a physiological (phosphate-buffered saline (PBS)) and in an acidic environment (HCl solution), mimicking the osteoclast-mediated bone resorption process, assessed by analysis of released calcium ions (degradation product of apatite-based biomaterials) (* statistically significant results compared to mat_HAP; *p* < 0.05, unpaired *t*-test).

The optimal biodegradation rate of implantable biomaterial is crucial, since it provides space for newly formed bone tissue, contributes to enhanced ion exchange, and protects against the collapse of the scaffold at the implantation site [25]. In this study, the biodegradation, in vitro, of the fabricated biomaterials, was evaluated under two different conditions: (1) biodegradation in phosphate-buffered saline (PBS), at physiological pH of 7.4, and (2) biodegradation in an acidic environment (HCl solution, pH 4.5), mimicking the osteoclast-mediated bone resorption process. The degree of biodegradation of bone scaffolds is shown in Figure 3 as the amount of released Ca^2+^ ions (degradation product of the ceramic) to the environment after incubation in PBS and HCl solutions. It was observed that the biodegradation of biomaterials in an acidic solution was faster, when compared to the PBS solution, at physiological pH. Surprisingly, mat_FAP revealed a higher degree of ceramic dissolution compared to mat_HAP, in both PBS and HCl solutions, which is not in agreement with the available scientific reports. Generally, mat_FAP released significantly higher amounts of Ca^2+^ ions compared to mat_HAP, with one exception, i.e., after 30 days of incubation of the biomaterials in the HCl solution, where HAP-based material released more calcium. The performed experiment showed that the fabricated bone scaffolds were prone to slow and gradual degradation, in both physiological conditions and an acidic environment imitating the osteoclast-mediated bone resorption process. It is worth emphasizing, that the slow and gradual biodegradation of implantable materials is crucial from the clinical point of view, because it allows for the progressive replacement of the bone scaffold with newly formed bone tissue [35]. Interestingly, according to the reports in the available literature, FAP is characterized by a lower bioresorption rate than HAP [23,36]. In this study, it was proved that biomaterial made of FAP, synthesized by using a combination of two methods: hyper-thermal hydrolysis and precipitation from aqueous solutions, was characterized by lower stability than the HAP-based sample, which indicated its higher bioabsorbability. Thus, it may be assumed that the fabrication method of the apatite-based biomaterials could have affected the properties of the bioceramics, and influenced their solubility and biodegradation behavior. Nevertheless, it should be noted that the good solubility of the FAP-based bone scaffold is a desired property, taking into account its clinical application as an implantable material.

### 2.4. Evaluation of Biological Response to Fabricated Biomaterials

**Figure 4:**
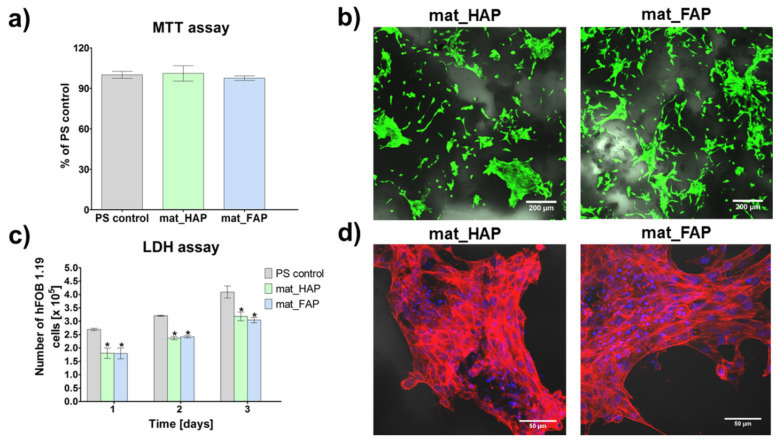
Biocompatibility assays with the use of normal human foetal osteoblast cell line (hFOB 1.19): (**a**) cytotoxicity assessment according to ISO10993-5 procedure by MTT assay, conducted with the use of 24 h biomaterial extracts (PS control—control cells exposed to culture medium incubated without biomaterial, in polystyrene wells, served as negative control of cytotoxicity); (**b**) confocal laser scanning microscope images, presenting live/dead staining of cells cultured on the surface of the biomaterials for 48 h (green fluorescence—live cells, red fluorescence—dead cells, Nomarski contrast was applied to visualize biomaterials, magnification 100×; scale bar = 200 µm); (**c**) cell proliferation assessment by total LDH assay (PS control—cells cultured in the polystyrene wells; * statistically significant results compared to PS control, *p* < 0.05, one-way ANOVA followed by Tukey’s test); (**d**) confocal laser scanning microscope images, presenting fluorescent staining of the cell cytoskeleton after 3-day cell culture on the surface of biomaterials (red fluorescence—actin filament, blue fluorescence—nuclei, magnification 200×; scale bar = 50 µm).

Apart from appropriate microstructural and physicochemical properties, bone implants should reveal some biological characteristics, such as biocompatibility and osteoconductivity, to accelerate bone tissue regeneration at the implantation site. Biocompatible biomaterials exert cellular response without toxic, immunogenic, or genotoxic effects. In turn, the ability of biomaterials to stimulate cell adhesion, proliferation, and bone extracellular matrix (ECM) synthesis by osteoblasts, is termed osteoconductivity [37,38]. In the presented study, the biocompatibility of the fabricated biomaterials was tested by an indirect method (MTT assay) and in direct contact with the scaffolds (live/dead staining of cells), using human foetal osteoblast cell line (hFOB 1.19), which is considered as an excellent cellular model, since it has many similarities to primary osteoblasts [38]. The performed MTT assay, showed that the fabricated biomaterials were non-toxic to human osteoblasts (Figure 4a). The cell viability of cells after exposure to 24 h extracts of mat_HAP and mat_FAP, was near 100%, compared to the polystyrene (PS) control. Additionally, live/dead staining of cells cultured on the surface of the biomaterials, showed a great number of viable cells (green fluorescence) with flattened morphology, indicating the non-toxicity of the scaffolds and good cell adhesion (Figure 4b).

The number of osteoblasts on the surface of the fabricated bone scaffolds was determined 1, 2, and 3 days after seeding, by total LDH assay (Figure 4c). The performed study showed that mat_HAP and mat_FAP supported cell proliferation at a similar level. On the third day of the experiment, the amount of hFOB 1.19 cells on the surface of the biomaterials was approximately 2-fold higher than on the first day. Additionally, fluorescent staining of the cell cytoskeleton after a 3-day culture on the surface of biomaterials, showed a high number of cells with extensive cytoskeleton structure, proving the osteoconductive properties of the scaffolds (Figure 4d). Interestingly, Borkowski et al. (2020) [34] showed that FAP granules sintered at 800 °C, were more favorable for the growth of mouse preosteoblasts than HAP granules. Likewise, Tredwin et al. (2014) [23] proved that FAP sintered at 600 °C, exhibited much more support for osteosarcoma cell proliferation than HAP. Nevertheless, the results of this study demonstrated that FAP- and HAP-based biomaterials exhibited similar biological properties. This may be a consequence of a much lower temperature (350 °C) applied during FAP and HAP production, that may affect cell behavior.

**Figure 5:**
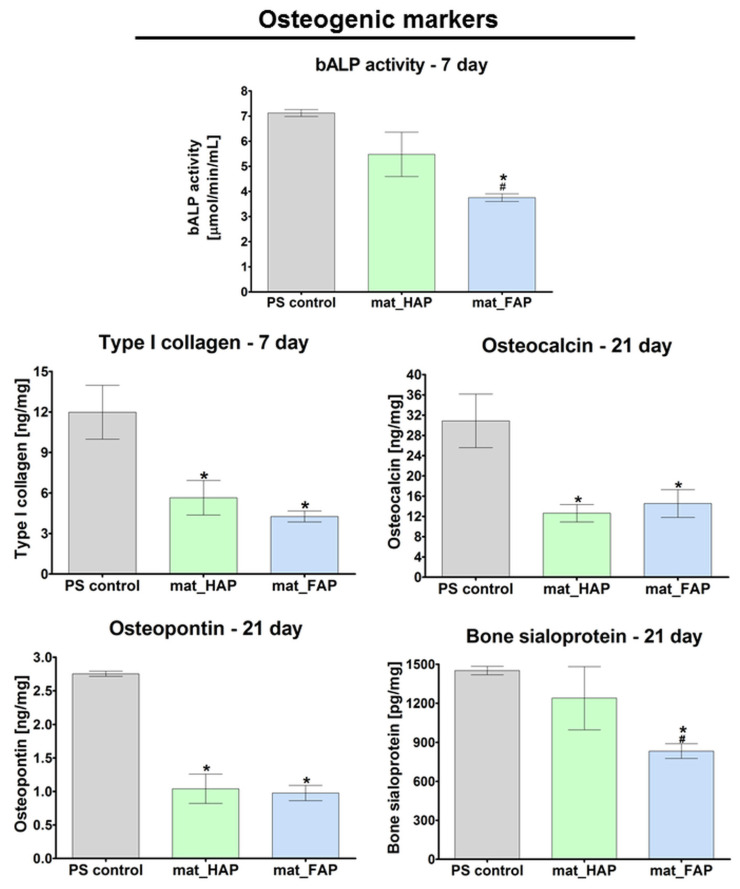
The level of osteogenic markers (bALP, type I collagen, bone sialoprotein, osteocalcin, and osteopontin), assessed by enzyme-linked immunosorbent assays (ELISAs) in human foetal osteoblasts (hFOB 1.19), grown on the biomaterials (PS control—cells cultured in the polystyrene wells; * statistically significant results compared to PS control, ^#^ statistically significant results compared to mat_HAP, *p* < 0.05, one-way ANOVA followed by Tukey’s test).

To guarantee good bone formation in vivo, stimulation of the osteogenic differentiation of cells (osteoblasts, osteoprogenitor cells, and mesenchymal stem cells) is a desirable characteristic of newly developed bone scaffolds. Osteogenic differentiation comprises three main stages, which are characterized by typical markers: (1) cell proliferation (RUNX2, Col I, OPN, fibronectin, low bALP activity), (2) ECM synthesis and maturation (high bALP activity, Col I, Osterix), and (3) ECM mineralization (OC, OPN, OCN, BSP, moderate bALP activity) [38,39,40]. Osteoblasts, mature bone-building cells, synthesize bone ECM proteins, mainly Col I, which is a framework supporting bone mineral deposition [25,38]. Moreover, active osteoblasts show high bALP activity, which provides great amounts of phosphates during ECM synthesis and maturation [38]. In this study, a quantitative evaluation of Col I synthesis and the bALP activity in hFOB 1.19 cells, after a 7-day culture on the surface of the fabricated biomaterials, was carried out (Figure 5). It was observed that cells cultured on the surface of mat_HAP exhibited significantly higher bALP activity compared to cells cultured on the mat_FAP.

**Figure 6:**
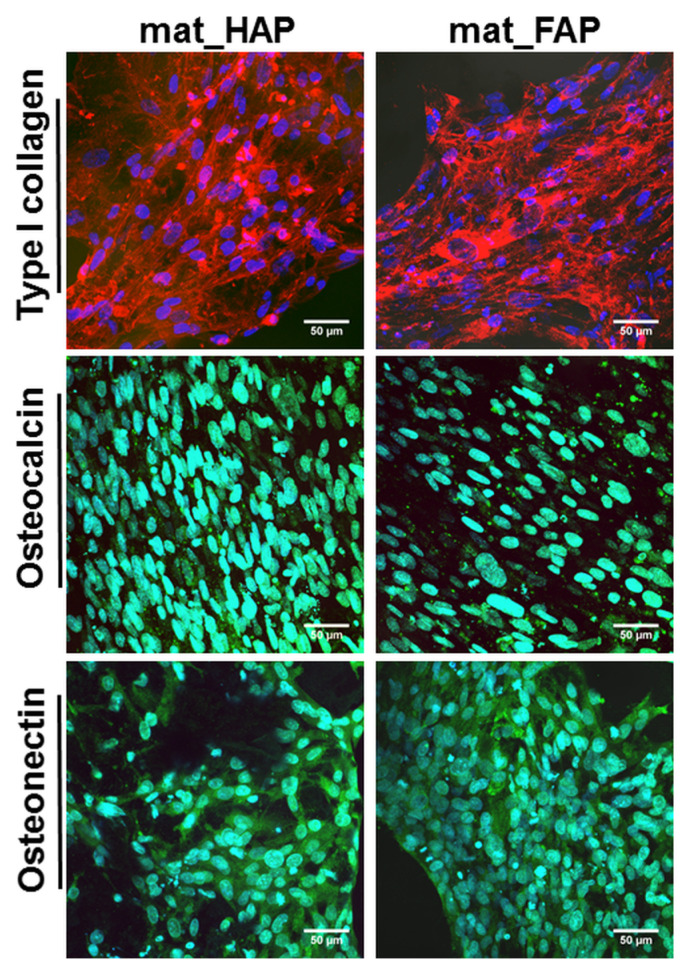
Confocal laser scanning microscope images presenting immunofluorescent staining of type I collagen (red fluorescence), osteocalcin (green fluorescence), and osteonectin (green fluorescence) in the extracellular matrix of human foetal osteoblasts (hFOB 1.19), grown on the biomaterials for 21 days (blue fluorescence—nuclei, magnification 400×; scale bar = 50 µm).

ELISA did not reveal statistically significant results in the level of Col I production between the samples, which was confirmed by CLSM images, that presented great deposits of Col I protein on the surface of both biomaterials after 21 days of culture (Figure 6). Late markers of osteogenic differentiation (BSP, OC, and OPN) were evaluated in hFOB 1.19 cells, after 21 days of culture on the surface of the fabricated biomaterials (Figure 5). Additionally, qualitative evaluation of OC and OCN (another late osteogenic differentiation marker) was performed by immunofluorescent staining (Figure 6). Osteoblasts cultured on mat_HAP and mat_FAP, synthesized very similar amounts of OC and OPN (Figure 5). However, it was observed that hFOB 1.19 cells cultured on the mat_HAP surface, produced higher amounts of BSP, as compared to mat_FAP. In turn, the immunofluorescent staining of OC and OCN, in cells cultured on mat_HAP and mat_FAP, did not show noticeable differences in the intensity of fluorescence (Figure 6). Nevertheless, the results of this study are not consistent with the several studies found in the available literature, which have demonstrated the stimulatory effect of fluoride on bone formation [20,41,42,43]. For example, Mansoorianfar et al. (2020) [43] showed that F^−^ ions increased bALP activity. However, it should be noted that the osteogenic differentiation of cells cultured on the surface of bone scaffolds may be affected by various factors, e.g., chemical composition, porosity, topographical features, and stiffness of the biomaterials [40]. Borkowski et al. (2021) [19] investigated an elastic biomaterial made of β-1,3-glucan and FAP or HAP bioceramic. Although the FAP-based material was more favorable to cell adhesion than the HAP-containing biomaterial, there were no differences in the level of osteogenic markers (bALP, Col I, OC) produced by hFOB 1.19 osteoblasts cultured on the FAP/glucan material or the HAP/glucan one.

The levels of all osteogenic markers, apart from bALP and BSP determined for mat_HAP, were significantly higher for control osteoblasts cultured in a 2D system on a flat polystyrene well, compared to the cells grown on bone scaffolds. However, it should be noted that the cells cultured on the 3D biomaterials had a significantly longer lag (adaptation) phase compared to the 2D culture. Consequently, the cells on 3D scaffolds reveal inferior proliferation, metabolism, and biological activity compared to the osteoblasts grown on flat polystyrene surfaces, when these parameters are assessed at the same time intervals [44]. Moreover, cells on 3D scaffolds are characterized by different morphology than well-spread osteoblasts on flat surfaces [45]. All of the above-mentioned factors are normal phenomena, and were reflected in the results obtained from the proliferation and osteogenic differentiation tests. Thus, the controls, in the form of osteoblasts cultured on 2D flat polystyrene wells, presented within the manuscript, actually served as positive controls of proliferation and osteogenic differentiation. It should be highlighted that the primary aim of the article was to compare osteoblast behavior on the surface of mat_FAP and mat_HAP.

**Figure 7:**
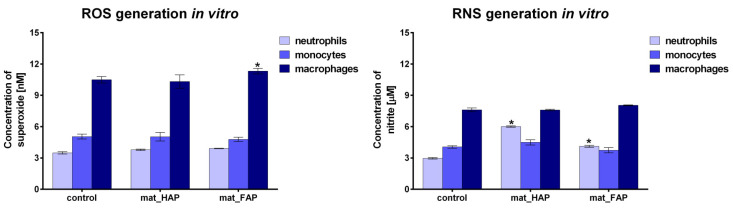
Evaluation of ROS/RNS generation by human immune cells (control—cells cultured without biomaterials; * statistically significant results compared to corresponding control cells, *p* < 0.05, unpaired *t*-test).

Bone scaffolds for regenerative medicine applications are expected to simultaneously promote osteoblast adhesion, proliferation, and differentiation, without induction of chronic inflammation after implantation, within the bone defect. During inflammation, activated immune cells have the ability to generate excessive amounts of ROS/RNS [38]. Within this study, the evaluation of ROS/RNS generation by human immune cells (neutrophils, monocytes, macrophages) was performed (Figure 7). Neutrophils and monocytes cultured in the presence of mat_HAP and mat_FAP did not generate higher amounts of ROS compared to control cells. In turn, macrophages cultured on mat_FAP produced slightly higher amounts of ROS, in comparison with corresponding control cells. It is worth noting, that excessive ROS generation may lead to oxidative damage of the bone scaffold at the implantation site, resulting in the failure of the biomaterial’s integration [46]. Furthermore, no excessive generation of RNS by monocytes and macrophages was observed. However, a significant increase in RNS production by neutrophils, cultured in the presence of the tested biomaterials, was observed. Importantly, Velard et al. (2009, 2010) [47,48] showed that HAP particles had the ability to activate neutrophils. Thus, the results obtained here are consistent with the available literature. Considering the performed experiments, it may be concluded that there is a very low risk that fabricated bone scaffolds would induce chronic inflammation after implantation within the bone defect. Nevertheless, further studies evaluating the inflammatory response to scaffolds are required.

## 3. Materials and Methods

### 3.1. Fabrication of the Apatite-Based Biomaterials

HAP and FAP were synthesized according to the procedure described in the Ukraine patent no. 56202, and with the method described previously [49], by using a combination of two synthesis methods: hyper-thermal hydrolysis (a dry method) and precipitation from aqueous solutions (a wet method). The initial components for the synthesis of apatites in chloride melt were mixtures of CaCO_3_, CaO, Ca(OH)_2_ (for HAP) or CaF_2_ (for FAP), and NaPO_3_. The synthesis was performed at a temperature of 350 °C. The Zeta potentials of the synthesized HAP (particle size = 581 nm) and FAP (particle size = 484 nm) were equal to −25.0 mV ± 0.9 and −33.0 mV ± 2.5, respectively [26]. The detailed chemical characterization, by FTIR analysis and Raman spectroscopy, and phase structure and lattice parameters by X-ray diffraction phase analysis, of the synthesized apatites, were described in previous studies [26,49].

Two types of apatite-based biomaterials, containing a chitosan–agarose matrix and 40% (*w*/*v*) of synthesized HAP (sample marked as mat_HAP) or FAP (sample marked as mat_FAP), were produced in accordance with the procedure described in the Polish patent no. 235822, and with the method described previously [24]. In brief, 2% (*w*/*v*) chitosan (50–190 kDa molecular weight, Sigma-Aldrich Chemicals, Warsaw, Poland), 5% (*w*/*v*) agarose (gel point 36 ± 1.5 °C, Sigma-Aldrich Chemicals, Warsaw, Poland), and the appropriate apatite, were suspended in acetic acid solution (Avantor Performance Materials, Gliwice, Poland) and mixed. Next, sodium bicarbonate (Sigma-Aldrich Chemicals, Warsaw, Poland) was added as a gas-foaming agent. The obtained paste was put in the molds and subjected to heating (95 °C). Then, samples were cooled, frozen, and lyophilized. The resultant scaffolds were immersed in 1% (*w*/*v*) sodium hydroxide solution (Avantor Performance Materials, Gliwice, Poland), rinsed with deionized water, and air-dried.

### 3.2. Microstructure Characterization

Visualization of the biomaterial microstructure was performed by a stereoscopic microscope (Olympus SZ61TR, Olympus Polska Sp. z o. o., Warsaw, Poland) and a scanning electron microscope (SEM, JEOL JCM-6000Plus, Japan). The porosity of the biomaterials was determined by micro-computed tomography—microCT (Xradia 510 Versa, Carl Zeiss X-ray Microscopy, Inc., Dublin, CA, USA), with a voxel resolution of 12 μm. To determine the pore diameter, as well astotal, open and closed porosity, the set of images (a total of 1601 images) was reconstructed into cross-section images of the biomaterials, using the Reconstructor software version 16.1 (Carl Zeiss X-ray Microscopy, Inc., Dublin, CA, USA), and then analyzed using the CTAnalyser software version 1.22 (Bruker microCT, Kontich, Belgium).

### 3.3. Compression Test

The compressive strength and Young’s modulus values of the biomaterials (9 mm in diameter and 9 mm in length) were measured using an Autograph AG-X plus (Shimadzu Corp. Kioto, Japan) testing machine, under the following parameters: crosshead moving speed of 5 mm/min, load cell accuracy of 0.1 N. The compressive stress was estimated to be 30% of strain.

### 3.4. Bioactivity Assessment

Determination of the apatite-forming ability of the fabricated biomaterials was performed in accordance with the ISO 23317:2012 procedure, and with the method described previously [24]. Briefly, the samples were incubated in a simulated body fluid (SBF) at 37 °C, for 28 days. After 28 days of incubation, apatite crystals that were formed on the surfaces of the biomaterials were visualized using SEM (Zeiss ULTRA plus) equipped with an Octane Pro EDS detector (EDAX) (Carl Zeiss Microscopy, LLC, White Plains, NY, USA). The presence of apatite precipitates on the surfaces of the samples was confirmed by EDS data, by calculation of the Ca/P atomic ratio. Additionally, during the incubation of the fabricated scaffolds in SBF, their samples were collected at determined intervals, to estimate the changes in concentrations of calcium (Ca^2+^) and phosphate (HPO_4_^3−^) ions. The Ca^2+^ and HPO_4_^3−^ concentrations were determined by a colorimetric method, using commercially available kits (BioMaxima, Lublin, Poland).

### 3.5. Biodegradation Assessment

An in vitro biodegradation assay was conducted in phosphate-buffered saline solution (PBS, pH 7.4, Sigma-Aldrich Chemicals, Warsaw, Poland), mimicking a physiological environment, and in an acidic solution (HCl solution, pH 4.5, Avantor Performance Materials, Gliwice, Poland), mimicking the osteoclast-mediated acidification during the bone resorption process. In brief, biomaterials weighing 50 mg ± 1 mg, were immersed in 5 mL of biodegradation solution and incubated at 37 °C, for 30 days. The biodegradation behavior of the biomaterials was determined by measurement of Ca^2+^ ion concentration (degradation product) by a colorimetric method, using a commercially available kit (Biomaxima, Poland).

### 3.6. Cell Culture Experiments

Normal human foetal osteoblast cell line (hFOB 1.19, ATCC-LGC Standards, Teddington, UK) was used, to assess the cytotoxicity, cell proliferation, and osteogenic differentiation on the surface of the fabricated apatite-based biomaterials. hFOB 1.19 cells were cultured in a 1:1 mixture of DMEM/Ham’s F12 medium without phenol red (Sigma-Aldrich Chemicals, Warsaw, Poland), with 10% foetal bovine serum (Pan-Biotech GmbH, Aidenbach, Bavaria, Germany), 100 U/mL penicillin, 100 μg/mL streptomycin, 300 μg/mL G418 (Sigma-Aldrich Chemicals, Warsaw, Poland), and maintained at 34 °C, with a 5% CO_2_ in air atmosphere. Osteogenic differentiation experiments were performed by cell culture, in osteogenic medium containing complete culture medium supplemented with 50 µg/mL ascorbic acid, 10 mM β-glycerophosphate, and 10^−8^ M dexamethasone (Sigma-Aldrich Chemicals, Warsaw, Poland). During osteogenic differentiation, the cells were cultured at 37 °C, with a 5% CO_2_ in air atmosphere.

In turn, evaluation of ROS and RNS generation in response to the fabricated biomaterials was performed by using immune cells (neutrophils, monocytes, and monocyte-derived macrophages) derived from human peripheral blood (informed consent was obtained from the volunteers; the Bioethics Committee approval no. KE-0254/187/10/2022). Isolation of neutrophils and monocytes was performed according to the previously described procedure [50,51]. Induction of monocytes differentiation towards mature macrophages was performed by adding 25 ng/mL macrophage colony-stimulating factor (M-CSF, Sigma-Aldrich Chemicals, Poland), followed by 5 days of culture at 37 °C, with a 5% CO_2_ in air atmosphere.

#### 3.6.1. Cytotoxicity Assessment

The cytotoxicity assessment was conducted by MTT assay, according to the ISO 10993-5 standard, using extracts of the biomaterials prepared in accordance with the ISO 10993-12 standard. In brief, biomaterial extracts were prepared by immersion of 100 mg of the biomaterial in 1 mL culture medium, and incubation at 37 °C, for 24 h. Culture medium incubated without biomaterial, in polystyrene wells, served as a negative control of cytotoxicity (sample marked as PS_control). The hFOB 1.19 cells were seeded into 96-multiwell plates, at a concentration of 2 × 10^4^ cells/well, and cultured at 34 °C, for 24 h. Subsequently, the medium was replaced with 100 μL of appropriate extracts of the biomaterial and the cells were cultured for the next 24 h. An MTT colorimetric assay (Sigma-Aldrich Chemical, Warsaw, Poland) was performed to evaluate cell viability, as described previously [52]. The obtained MTT results were shown as the percentage of OD value obtained with the negative control.

Additionally, a cytotoxicity assessment in direct contact was performed. The hFOB 1.19 were seeded directly onto the biomaterials at a concentration of 5 × 10^4^ cells per sample. After 48 h of culture, cells were stained using the Live/Dead Double Staining Kit (Sigma-Aldrich Chemicals, Warsaw, Poland), according to the manufacturer’s procedure, and visualized by confocal laser scanning microscope (CLSM, Olympus Fluoview equipped with FV1000, Olympus Polska Sp. z o. o., Warsaw, Poland).

#### 3.6.2. Evaluation of Cell Proliferation

The hFOB 1.19 cells were seeded directly onto the biomaterials at a concentration of 1 × 10^5^ cells per sample. After 1, 2, and 3 days of culture, the number of cells was determined by using the total activity of the lactate dehydrogenase assay (LDH, Sigma-Aldrich Chemicals, Warsaw, Poland), after cell lysis. The LDH assay was performed in accordance with the manufacturer’s procedure. The cell number was estimated from the calibration curve, made for known concentrations of hFOB 1.19 cells. Additionally, after 3 days of cell culture on the surface of the biomaterials, visualization of osteoblasts was carried out, using fluorescent staining and CLSM observation. Before visualization, cells were subjected to fixation and permeabilization, by using paraformaldehyde and Triton X-100 (Sigma-Aldrich Chemicals, Warsaw, Poland), respectively. Then, the cells were dyed with AlexaFluor635-conjugated phallotoxin (Invitrogen, Carlsbad, CA, USA), that stains actin filaments, and DAPI (Sigma-Aldrich Chemicals, Warsaw, Poland), that stains nuclei.

#### 3.6.3. Osteogenic Differentiation Assessment

The hFOB 1.19 cells were seeded directly onto the biomaterials at a concentration of 2 × 10^5^ cells per sample, and cultured at 37 °C, in an osteogenic medium, for 21 days. Every 3rd day, half of the osteogenic medium was replaced with a fresh portion. To assess the osteogenic differentiation process in vitro, osteogenic markers (type I collagen (Col I), bone sialoprotein (BSP), osteocalcin (OC), osteopontin (OP)) were estimated in cell lysates, using appropriate enzyme-linked immunosorbent assays (ELISAs) (EIAab ELISAs kit, Wuhan, China). The cell lysates were obtained by two freeze-thaw cycles and then a sonication process, at 30% amplitude, as described previously [53]. The ELISA results were normalized to the total cellular proteins estimated by the BCA Protein Assay Kit (ThermoFisher Scientific, Waltham, MA, USA), and expressed as ng or pg of the osteogenic marker per mg of total cellular proteins. Moreover, bone alkaline phosphatase (bALP) activity was determined colorimetrically by an Alkaline Phosphatase Assay Kit (Sigma-Aldrich Chemicals, Warsaw, Poland). The test was performed according to the manufacturer’s protocol. Additionally, after 21 days of osteoblast culture on the surface of the biomaterials, immunofluorescent (IF) staining of osteogenic markers (Col I, OC, and osteonectin (OCN)) was performed, in accordance with the procedure described previously [45].

#### 3.6.4. Evaluation of ROS/RNS Generation by Immune Cells

Immune cells (neutrophils, monocytes, and macrophages), at a density of 1 × 10^6^, were seeded onto the surface of the fabricated biomaterials, and into the wells of 24-multiwell plates without biomaterials, which served as controls. After 24 h culture of neutrophils and monocytes on the fabricated biomaterials, and after 5 days of culture in the case of macrophages, ROS/RNS generation was determined. Evaluation of superoxide ($O_{2}^{-})$ and nitrite (${\mathrm{NO}}_{2}^{-})$ generation was performed according to the previously described colorimetric methods [50,51], by using nitroblue tetrazolium solution and the Griess reaction, respectively.

### 3.7. Statistical Analysis

In this study, experiments were performed at least in triplicate (n ≥ 3). The data were shown as mean values ± SD and were evaluated for normal distribution using the Shapiro–Wilk test. Statistically significant results between samples were considered at *p* < 0.05 and were estimated using unpaired *t*-test or one-way ANOVA, followed by Tukey’s test (GraphPad Prism 8.0.0 Software).

## 4. Conclusions

The results obtained within this study, showed that HAP- and FAP-based bone scaffolds are characterized by a macroporous microstructure, with a network of interconnected pores and compressive strength comparable to cancellous bone. The novel biomaterials are biodegradable under physicochemical conditions (pH 7.4) and in acidic environments (pH 4.5), mimicking the osteoclast-mediated bone resorption process. Moreover, the scaffolds are characterized by high biocompatibility, osteoconductive properties, and have the ability to induce apatite formation on their surfaces, indicating bioactive properties. Moreover, HAP- and FAP-based bone scaffolds support osteogenic differentiation and do not induce generation of excessive amounts of ROS/RNS by immune cells, increasing their potential in biomedical applications.

Nevertheless, the performed comprehensive comparative study on HAP- and FAP-based bone scaffolds did not show significant differences between the biomaterials. However, it may be concluded that FAP is a better bioceramic component for the production of this specific bone scaffold (according to Polish patent no. 235822), due to a higher porosity and primary bioabsorbability of the FAP-based biomaterial compared to HAP-containing sample, shown in this study. A good bioabsorbability of implantable materials is a very important property from the clinical point of view, because it allows for progressive replacement of the bone scaffold with newly formed bone tissue. However, it is worth taking into account that the applied production method of the bioceramic-based biomaterials could have affected the physicochemical and biological properties of the apatite component. Thus, FAP is a better bioceramic for the production of this particular bone scaffold. It may reveal different properties after incorporating it as a component in other scaffolds. Therefore, each biomaterial should be tested and considered individually, prior to biomedical application.

## 5. Patents

The methods for the synthesis of HAP and FAP are protected by Ukraine patent no. 56202. The method for the production of the apatite-based scaffolds is protected by Polish patent no. 235822.

## Figures and Tables

**Table 1:** Evaluation of porosity and mechanical properties of fabricated scaffolds.

Porosity [%]	mat_HAP	mat_FAP
Total	37.52 ± 1.87	47.52 ± 2.95 *
Closed	30.43 ± 2.59	36.06 ± 4.62 *
Open	7.09 ± 2.34	11.46 ± 6.35 *
**Mechanical Parameters [MPa]**	**mat_HAP**	**mat_FAP**
Compressive strength	3.01 ± 0.59	2.58 ± 0.75
Young’s modulus	19.14 ± 13.45	19.08 ± 10.38

* Statistically significant results compared to mat_HAP, *p* < 0.05, unpaired *t*-test.

## Data Availability

The raw/processed data required to reproduce these findings can be obtained from the corresponding author (paulina.kazimierczak@umlub.pl) upon reasonable request.
